# Determination and Identification of Nicarbazin, Measured as 4,4′-Dinitrocarbanilide (DNC), in Chicken Tissues by Liquid Chromatography With Tandem Mass Spectrometry: Final Action 2013.07

**DOI:** 10.1093/jaoacint/qsac011

**Published:** 2022-02-02

**Authors:** Sharon L Brunelle, Robert A LaBudde, Kimberly Lombardi, Clive Ward

**Affiliations:** Brunelle Biotech Consulting, 6620 NW Burgundy Dr., Corvallis, OR 97330, USA; Least Cost Formulations, Ltd, 824 Timberlake Dr., Virginia Beach, VA 23464, USA; Research Breakthrough Innovation, Elanco Animal Health, 2500 Innovation Way, Greenfield, IN 46140, USA; Bioanalytics, Global Development Operations, Elanco Animal Health, 245 Western Road, Kemps Creek, NSW 2178, Australia

## Abstract

**Background:**

AOAC Method **2013.07** was adopted as First Action in 2013. Since then, the method has been used in numerous residue depletion studies with favorable comments from analysts.

**Objective:**

To analyze data from residue depletion studies to support Final Action status.

**Method:**

Ten residue depletion studies were conducted during May 2014 through May 2019. For each study, harvested incurred tissues were analyzed for nicarbazin using AOAC Method **2013.07** in 1 of 4 laboratories. Each analytical run included one or more fortified quality control test portions. The data from these known fortified matrix test portions were analyzed for reproducibility and repeatability.

**Results:**

For muscle tissues, relative recovery was 90.4% (95% CI 83.8 to 97.5); RSD_r_ was 5.4% (95% CI 3.8 to 9.2); and RSD_R_ was 7.9%. In the liver, values were 94.5% (95% CI 91.1 to 98.0), 5.8% (95% CI 4.1 to 9.9), and 6.8%, respectively. In the kidney, values were 91.5% (95% CI 85.3 to 98.1), 5.2% (95% CI 3.7 to 8.8), and 9.0%, respectively. In skin with adhering fat, values were 94.5% (95% CI 89.2 to 100.1), 8.9% (95% CI 6.3 to 15.1), and 8.9%, respectively. In all cases, repeatability and reproducibility were within acceptable limits.

**Conclusions:**

The data and positive feedback support the transition of AOAC Method **2013.07** from First Action to Final Action.

**Highlights:**

Final action status is supported by data collected during routine use of the method rather than a traditional multi-laboratory collaborative study. Data were subjected to statistical analysis using the pC-metamer, and then transformed back to the traditional C-metamer.

Nicarbazin is used as an aid to prevent cecal infection with intracellular parasites, known as Coccidia, in broiler chickens by administration through medicated feed (1). Approvals for nicarbazin alone or in combination with ionophores (monensin or narasin) exist for use in broilers and turkeys globally (registrations include regions such as the United States, the European Union, Australia, New Zealand, Malaysia, and Canada); however, due to a reduction in reproductive capacity, nicarbazin is not approved for laying hens. Nicarbazin is also approved for control of feral pigeon populations in urban areas (2). Nicarbazin is composed of equimolar amounts of 4,4ʹ-dinitrocarbanilide (DNC) and 2-hydroxy-4,6-dimethyl pyrimidine (HDP), with DNC being the more persistent residue (3) and thus the marker residue used for screening of edible poultry tissues. As a result of the wide reach of global approvals for nicarbazin, a robust and reproducible method to enable surveillance across the globe is of significant importance to enable trade and ensure a safe food supply.

Codex Alimentarius and EU Maximum Residue Limits (MRLs) and U.S. tolerances for nicarbazin, measured and expressed as DNC concentrations, in various chicken tissues are presented in Table 1. The U.S. tolerances were updated from 4000 to 52* *000 µg/kg in liver tissue in 2018, which resulted in a much higher range than the method was originally intended to cover. A dilution procedure was described in the method to accommodate the expanded range for U.S. tolerance, thereby allowing for continued applicability of this method for use in monitoring nicarbazin residues in the United States.

**Table 1.** Maximum residue limits (EU and Codex) and tolerances (U.S.) for Nicarbazin in chicken tissues measured and expressed as DNC concentration

Tissue	Nicarbazin (measured and expressed as DNC)		
	Codex MRL, µg/kg	EU MRL, µg/kg	U.S. tolerance, µg/kg
Liver	200	15* *000	52* *000
Kidney	200	6000	NA
Muscle	200	4000	NA
Skin/Fat	200	4000	NA
Eggs	NA^a^	NA	NA

a While there is no MRL in eggs, there is a “maximum limit” of 100 µg/kg.

The AOAC Expert Review Panel (ERP) for Veterinary Drug Residues granted First Action status to AOAC Method **2013.07** in 2013 (4). This report presents data to support the recommendation that the method be ascribed Final Action status. 

## Analysis of Physiologically Incurred Nicarbazin Drug Residues in Chicken and Eggs Conducted in Four Laboratories

###  

Ten residue depletion studies were conducted to generate data to support drug registration for nicarbazin as a feed additive for chicken. These studies included administering label doses of nicarbazin to the food animals of interest (chicken) and withdrawing the animals following the last drug administration to establish withdrawal periods to demonstrate that when nicarbazin is administered to chicken as per label instructions, nicarbazin drug residues will not be detected above the regulatory safe limits such as MRLs/tolerances established for chicken tissues and eggs.

A fully validated analytical method meeting the requirements of ISO/IEC 17025:2017 (5) and Veterinary International Conference on Harmonization (VICH) criteria (6) was required to be used for the analysis of tissue samples generated in a residue depletion study to support product registration. In that respect, AOAC Method **2013.07**, which had been validated under single laboratory conditions and had been accorded AOAC First Action status, was used to measure the concentration of the nicarbazin residues in chicken tissues harvested from experimental chicken over the course of the withdrawal period for each of these 10 studies.

This innovative approach was used in place of the traditional approach, where 10 to 12 laboratories were required to participate in a multi-laboratory study to generate reproducibility precision data.

Data are presented in this article to support Final Action recommendation for AOAC Method **2013.07.** The data include standard curves, method quality control (QC) test portion results, nicarbazin residues measured in the residue depletion study, and feedback on the use of the method from the laboratories that conducted the drug residues analysis.

The four laboratories that analyzed the nicarbazin drug residues in the physiologically induced incurred tissue samples and in eggs were the Eurofins Food Integrity and Innovation Laboratory (Greenfield, IN, USA), Harlan Laboratories S.A. (Barcelona, Spain), Labfor Análises Laboratoriais, Ltda. (São Paulo, Brazil), and the Charles River Laboratories Edinburgh Ltd. (East Lothian, UK). The studies were randomly assigned numbers 1–10 for the purposes of this report. *Official Method*^SM^**2013.07** was followed as written with no deviations reported.

      **AOAC *Official Method***^**SM**^**2013.07** **Determination and Identification of Nicarbazin, Measured as**  **4,4′-dinitrocarbanilide (DNC)**, **in Chicken Tissues by**  **Liquid Chromatography With Tandem Mass Spectrometry**     **First Action 2013**

        **Final Action 2021**

[Applicable for the determination and identification of nicarbazin (measured and expressed as 4,4′-dinitrocarbanilide; DNC) in chicken liver, kidney, muscle, and skin with adhering fat tissues, and in eggs.]

*Caution:* Solvents employed are common use solvents and reagents. Refer to adequate manuals or safety data sheets to ensure that the safety guidelines are applied before using chemicals. Store in a flammable liquid storage cabinet. Harmful if inhaled, swallowed, or absorbed through the skin. Use appropriate personal protective equipment such as a lab coat, safety glasses, rubber gloves, and a fume hood. Dispose of all materials according to federal, state, and local regulations.

### A. Principle

Poultry tissue is cryogenically homogenized with solid sodium sulfate, and then extracted twice with acetonitrile. Extracts are combined, filtered, and diluted accordingly based on the regulatory limits being targeted and the working concentrations of the standards used for LC-MS/MS analysis. Identification is accomplished by comparing the product ions measured in the samples to those present in the standard injections in mass and relative intensity, and comparison of chromatographic retention times between samples and standards. Nicarbazin determination and identification is based on the DNC portion of the molecule as are the regulatory limits and tolerances. Concentrations are determined by LC-MS/MS using a matrix-matched standard curve and DNC-d_8_ internal standard added prior to test portion extraction.

### B. Apparatus

- *Volumetric pipettes*.—Class A, glass, assorted sizes.
- *Positive displacement pipettes*.—Gilson, Inc. (Middleton, WI) Model No. M100 Part No. F148504 (10–100 µL), Model No. M250 Part No. F148505 (50–250 µL), and Model No. M1000 Part No. F148506 (100–1000 µL).
- *Volumetric flasks*.—Class A, glass, assorted sizes.
- *Analytical balances*.—Sensitive to at least 0.01 and 0.00001 g.
- *Actinic glassware*.—Or glassware covered with aluminum foil.
- *Spatulas*.—Stainless steel or Teflon-coated.
- *Glass bottles*.—Corning (Corning, NY), 1 or 2 L.
- *Graduated cylinders*.—glass, assorted sizes.
- *Magnetic stirrer and Teflon-coated stir bars*.
- *Cryogenic grinding and homogenization equipment.*—Foss (Eden Prairie, MN) or Robot Coupe (Ridgeland, MS) grinder or a Waring blender or equivalent.
- *Multi-tube vortex mixer*.—VWR (Radnor, PA) Model No. DVX-2500.
- *Polypropylene centrifuge tubes*.—50 mL conical with closures.
- *Centrifuge*.—Refrigerated (temperature controlled), capable of 3000 rpm and 5°C.
- *Transfer pipets*.—Disposable.
- *Filters*.—Pall Gelman (Ann Arbor, MI) Acrodisc™, PTFE, 13 mm, 0.45 µm.
- *HPLC vials with caps*.
- *LC-MS/MS*.—AB Sciex (Framingham, MA) API4000, TurboIonSpray^®^ probe, Analyst^®^ software.
- *HPLC pump and autosampler*.
- *Chromatographic column*.—Restek (State College, PA) Aqueous C_18_, 3 µm, 2.1 × 50 mm (Part No. 9178352 for 3 µm particle size or Part No. 9178552 for 5 µm particle size).

### C. Materials and Reagents

- *Methanol (MeOH)*.—HPLC grade.
- *Water (H_2_O)*.—HPLC grade or distilled, deionized.
- *Acetonitrile (ACN)*.—HPLC grade.
- *Sodium sulfate (Na_2_SO_4_)*.—Anhydrous granular, Certified ACS.
- *Ammonium acetate (NH_4_OAc)*.—Certified ACS.
- *Formic acid (FA)*, concentrated.—Certified ACS.
- *N, N′* *-dimethyl formamide (DMF)*.—Certified ACS.
- *Nicarbazin reference standard*.—Eli Lilly and Company (Indianapolis, IN). Composed of equimolar quantities of DNC and 2-hydroxy-4,6-dimethyl pyrimidine (HDP). When ordered from Eli Lilly and Co., the order will be accompanied by a certificate of analysis that gives details on the DNC purity. Store at 15 to 30°C. Consult the MSDS for safety and handling information.
- *DNC-d_8_ internal standard*.—Sigma-Aldrich (St. Louis, MO), Part No. 34214.

### D. Preparation of Reagents and Standards

- *Mobile phase solution A*.—To 1000 mL H_2_O, add 1.0 mL FA and 0.38 ± 0.04 g NH_4_OAc and mix thoroughly.
- *Mobile phase solution B*.—To 1000 mL MeOH, add 1.0 mL FA and 0.38 ± 0.04 g NH_4_OAc and mix thoroughly.
- *Nicarbazin stock standard solution (1000 µg/mL DNC component)*.—Accurately weigh 141.4 mg nicarbazin reference standard, equivalent to about 100.0 mg DNC when compensated for purity, and transfer to a 100 mL volumetric flask. Dissolve with sonication (approximately 10 min) and dilute to volume with DMF. Mix thoroughly.
- *Nicarbazin intermediate standard solution (10 µg/mL DNC component)*.—Make a 100-fold dilution of the nicarbazin stock standard solution (1000 µg/mL DNC) with ACN.
- *Nicarbazin standard curve solutions.*—Make dilutions from the nicarbazin intermediate standard solution (10 µg/mL DNC) with ACN to prepare a standard curve of 25, 50, 125, 500, 1250, and 2500 ng/mL.
- *DNC-d_8_ internal standard stock solution (1.0 mg/mL)*.—Using DMF, dissolve and transfer the 10 mg vial of DNC-d_8_ standard into a 10 mL volumetric flask. Dilute to volume with DMF and mix thoroughly.
- *DNC-d_8_ internal standard solution (1.0 μg/mL)*.—Make a 1000-fold dilution of the DNC-d_8_ stock solution with ACN.

*Note*: Different volumes of equivalent concentrations may be substituted.

*Note*: Store all stock standards and standard solutions at room temperature protected from light. Stock standards are stable for 3 months and standard solutions for 14 days under these conditions.

### E. Sample Preparation

- *Homogenization and storage of samples.*—Initial processing includes grinding or blending of the tissues using cryogenic grinding to produce homogeneous samples. Cryogenic grinding is carried out by freezing the tissue with liquid nitrogen or dry ice and then grinding into a fine powder using a Foss or Robot Coupe grinder or a Waring blender. This process is used to produce a very fine homogeneous powder of the tissue for analysis. Grind a minimum 500 g sample of tissue when possible. Subsamples of 5.00 ± 0.05 g tissue (1.00 ± 0.05 g for kidney) may be weighed into 50 mL polypropylene tubes and frozen. This will minimize tissue exposure to multiple freeze/thaw cycles. Store all tissues at freezer temperatures (−20°C or below) when not processing or subsampling. It is advisable to store fortified samples of all tissues with experimental samples to verify storage stability.
- *Preparation of quality control (QC) and negative control (NC)tissues*.—On the day of analysis, prepare at least seven NC matrix samples and a matrix sample fortified at MRL or tolerance [QC sample, *see* **E**(**c**)*(3)*]. Process QC and NC samples as indicated in **E**(**c**).
- *Tissue extraction*.—Poultry muscle, liver, kidney, skin with adhering fat, and eggs:
  1. Accurately weigh 5.00 ± 0.05 g (1.00 ± 0.05 g for kidney) of a representative ground sample of frozen or partially thawed sample into a 50 mL conical polypropylene centrifuge tube.
  2. Fortify all samples with 200 μL (40 μL for kidney) of the 1.0 μg/mL DNC-d_8_internal standard solution.
  3. Fortify QC samples with nicarbazin (based on DNC content and purity) at MRL or tolerance.
  4. Add 10 ± 1 g of anhydrous sodium sulfate to each tissue sample (2.0 ± 0.2 g for kidney).
  5. Thoroughly incorporate the sodium sulfate into the tissue sample using a stainless steel or disposable wooden spatula to generate a crumbly or pasty tissue homogenate.
  6. Add 20 mL ACN and mix using a multi-tube vortex mixer for 30 min.
  7. Centrifuge the sample at approximately 3000 rpm (RCF = approximately 2025 × *g*) for 10 min.
  8. Decant the supernatant into another graduated vessel (50 mL conical centrifuge tube or mixing cylinder).
  9. Re-extract the tissue pellet following steps **E**(**c**)*(6–8)* and combine the supernatants.
  10. Add 1.0 mL of nicarbazin standard curve solutions to each of six NC extracts to prepare the matrix-matched curve. Final concentrations are 0.5, 1.0, 2.5, 10, 25, and 50 ng/mL.
  11. Adjust all samples to 50mL final volume with ACN and mix thoroughly.
  12. Filter the samples into LC vials for analysis.

### F. Determination

- *LC operating conditions*.—(*Note*: These guidelines may be modified to obtain the desired chromatography.) Column temperature, 30°C; flow rate, 0.4 mL/min; autosampler temperature, ambient; injection volume, 10 µL; run time, 12 min; gradient, 0–2 min 0% mobile phase B, 2–3 min 0–80% mobile phase B, 3–6 min 80–100% mobile phase B, 6–8 min 100% mobile phase B, 8–8.2 min 100–0% mobile phase B. *See*Figure 2013.07A for representative chromatogram.
- *MS/MS operating conditions*.—(*Note*: Equivalent equipment can be substituted. The MS parameters provided are suggested values for the API 4000 instrument. For optimal analysis, MS parameters should be obtained by instrument tuning.) Instrumentation, AB SCIEX API 4000 Triple Quadrupole Mass Spectrometer; operating mode, negative ion, selected reaction monitoring [*Note*: Analyst software denotes this as multiple reaction monitoring (MRM)]; determinative transition, *m/z* 301.0→136.7; identification transition, *m/z* 301.0→106.9; internal standard transition, *m/z* 308.7→140.6.
- *Mass spectrometer compound-specific parameters*.—DNC, Q1 mass 301.0 amu, Q3 mass 136.7 amu, collision energy −16 V, collision cell exit potential −11 V, entrance potential −6 V; DNC, Q1 mass 301.0 amu, Q3 mass 106.9 amu, collision energy −48 V, collision cell exit potential −7V, entrance potential −4 V; DNC-d_8_, Q1 mass 308.7 amu, Q3 mass 140.6 amu, collision energy −16 V, collision cell exit potential −7 V, entrance potential −6 V.
- *Mass spectrometer non-* *compound-specific parameters*.—Ion source, turbospray; resolution (Q1 and Q3), unit; curtain gas (CUR), 20; ion spray (IS), −4500 V; collisional activated dissociation (CAD), 10; declustering potential (DP), −55 V; source temperature, 550°C.
- *System suitability.*—A sufficient number of injections should be made of the final NC extract containing the internal standard such that the response of the internal standard has stabilized. It is left to the discretion of the analyst to determine when the *y*-axis response has stabilized. It may take anywhere from 5 to 10 injections for this to occur.
- *Quantitative analyses.*—
  1. Make single injections of the matrix-matched standard solutions, single injections of each sample extract solution, and then again single injections of the matrix-matched standard solutions. *Note*: Standard injections at the beginning and end of the run can be made out of the same HPLC vial. It is recommended to not exceed 12 sample injections between injections of a standard curve.
  2. Measure the peak areas for DNC and DNC-d_8_ in the standard and sample solutions. Construct a 1/x weighted linear standard curve using determinative ion ratios of the standard responses (ratio of 301.0→136.7 to 308.7→140.6; DNC to DNC-d_8_) vs concentration. A 1/x^2^ weighting can be used if the residuals are smaller. From the standard curve, calculate the concentrations in ng/mL of each of the extracted samples.
  3. Using weight, volume, dilution from **F**(**f**)*(4)* if any, and concentrations from **F**(**f**)*(2)*, calculate the DNC concentration in the samples.
    DNC tissue concentration=µg/kg=[(A⋅B)/C]⋅D
    where A = sample concentration from standard curve (ng/mL); B = extract volume (mL); C = weight of tissue sample (g); and D = dilution factor.
  4. If the determinative ion ratio exceeds the high end of the standard curve, the extracted sample should be diluted with control matrix extract and reinjected along with the standard curve.
    For the liver, if the tissue concentration is between 500 µg/kg (equivalent to the upper end of the standard curve) and 8000 µg/kg, the extracted sample should be diluted with control matrix extract and reinjected along with the standard curve.
    For the liver, if the tissue concentration of DNC exceeds 8000 µg/kg, then the original tissue sample should be diluted in negative control tissue (for example, 1 g sample tissue + 4 g control tissue) and reextracted.
- *Qualitative* *identification.*—Identification is accomplished by comparing the product ions measured in the samples to those present in the standard injections in both mass and relative intensity.
  1. Obtain the individual ion chromatograms for the product ions and ensure that the chromatographic retention times for the analytes are ±5% relative to the mean retention time of the appropriate analyte in the standard. Extracts may be reinjected if there has been a sudden shift in retention time during the batch analysis exceeding the 5% tolerance.
  2. Integrate the area of the DNC peak for each selective reaction monitoring (SRM) trace for the standards and samples. From the integrated area values for DNC, represent the determinative ion as 100% (*m/z* 301.0→136.7) and calculate the abundance of the identification ion (*m/z* 301.0→106.9) as a relative percentage for each standard and sample. Using the mean ion abundance percentages (IAP) of the standard solutions within a chromatographic run, calculate the U.S. acceptance range (7) as mean ± 10% arithmetic difference for the samples within that run. For example, at 20% mean IAP of standards the U.S. acceptance range would be 10–30% IAP for samples within that run. For the EU (8), the acceptance range is ± 40% relative to the mean IAP of standards. For example, at 20% mean IAP of standards, the EU acceptance range would be 12–28% for the samples within that run.
- *Standard curve acceptability criteria*.—The following criteria will be used for determining curve acceptability:
  1. Back-calculated accuracy for any standard curve point must be within ±15% of the theoretical value (±20% of the theoretical value at the lower limit of quantitation).
  2. Individual data points may be excluded in a given batch provided the curve maintains a minimum of five different concentrations and the standards bracket the QC and unknown test portions.
- *QC acceptability criteria.—*The following criteria will be used for determining QC acceptability:
  1. Determine recovery of the QC test portions as recovery = (concentration/actual fortification level) × 100.
  2. QC test portions must meet the recovery requirements [e.g., 70–110% at ≥10 µg/kg to <100 µg/kg from the Veterinary International Conference on Harmonization (VICH) Guideline (6)].

## Results and Discussion

### Standard Curves

**Figure 1.**
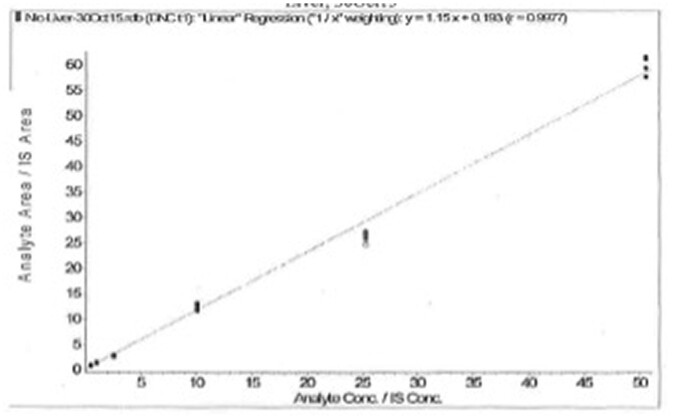
Laboratory 2 standard curve for liver. Regression with 1/x weighting; *y* = 1.15*x* + 0.193; r = 0.9977; r2 = 0.9954.

Matrix-matched standard curves were prepared and analyzed according to the method. A representative curve for chicken liver is shown in Figure 1. Weighting and regression data of representative standard curves from each laboratory for each matrix are summarized in Table 2. The standard curve acceptability criteria are found in Section F(g) of the method and include: back-calculated accuracy for any standard curve point must be within ±15% of the theoretical value (±20% of the theoretical value at the LLOQ); and individual data points may be excluded in a given batch provided the curve maintains a minimum of five different concentrations and the standards bracket the QC and unknown test portions.

**Figure 2013.07A.**
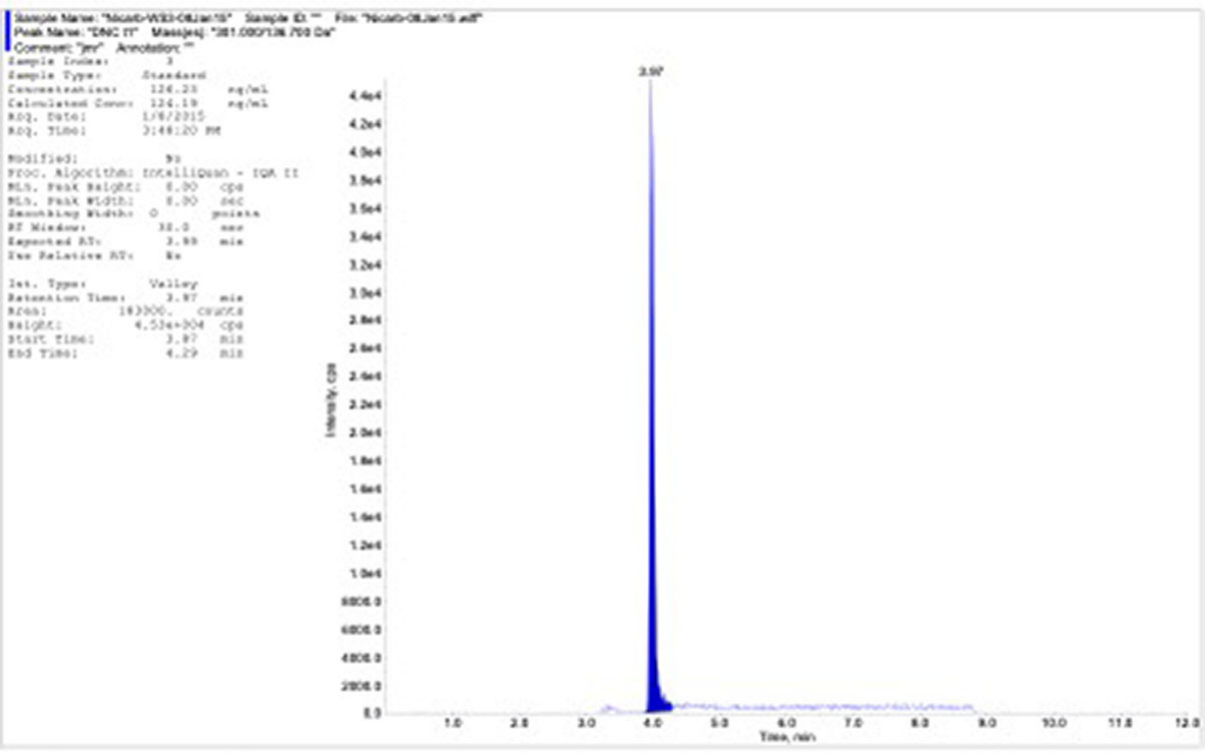
Representative chromatogram of chicken liver matrix-matched standard at 125 ng/mL.

**Table 2.** Regression analysis of representative matrix-matched calibrations curves

Laboratory	Matrix	Weighting	Slope	Intercept	Correlation coefficient (R^2^)
1	Muscle	1/x^2^	0.698	0.00649	0.995
	Skin w/Fat	1/x^2^	0.714	0.0279	0.996
	Liver	1/x^2^	0.679	0.00901	0.998
	Kidney	1/x^2^	0.777	0.00694	0.995
2	Muscle	1/x	1.14	0.0992	0.995
	Skin w/Fat	1/x	1.05	0.156	0.998
	Liver	1/x	1.15	0.193	0.995
	Kidney	1/x	4.68	0.193	0.992
3	Muscle	1/x	0.0541	0.000251	0.995
	Skin w/Fat	1/x	0.0535	0.000580	0.997
	Liver	1/x	0.0458	0.000247	0.998
	Kidney	1/x	0.0592	0.000277	0.998
4	Muscle	1/x	0.230	0.0162	0.997
	Skin w/Fat	1/x	0.248	0.0332	0.995
	Liver	1/x	0.241	0.00872	0.990
	Kidney	1/x	0.332	0.0775	0.991

In Laboratory 1, the following additional criteria were applied: the coefficient of correlation (r) should be ≥0.990; 75% of the calibration standards should comply with the back-calculated accuracy requirements; and the calibration curve must have at least one calibration standard at the highest level (ULOQ) and another at the lowest level (LLOQ).

In Laboratory 3, the following additional criteria were applied: the coefficient of correlation (r) should be ≥0.980; the data should pass the homoscedasticity test (Cochran’s test); the data should demonstrate little to no multicollinearity; and the data should show no autocorrelation.

In all cases, the applied criteria were met on each day of testing. It should be noted that the axes and slopes of the standard curves differ based on the concentration units used in the software for calculations. In some cases, the solution concentration (ng/mL) was used, and in other cases the corresponding tissue concentration (µg/kg) was used.

### Quality Control Test Portion Samples

QC test portions were prepared and analyzed according to the method, which requires at least one matrix-matched QC test portion fortified at the regulatory level (MRL or tolerance) for each tissue analyzed. QC acceptance criteria are found in Section F(h) of the method and include: the determination of the recovery of the QC test portions as Recovery = (concentration/actual fortification level)*100 and QC test portions must meet the recovery requirements [e.g., 70–110% at ≥10 µg/kg to <100 µg/kg from the VICH Guideline (6)].

U.S. Food and Drug Administration recovery requirements (5, harmonized with the EU and Japan) were followed and are shown in Table 3. Additional QC sample criteria from the Bioanalytical Method Validation Guidance for Industry (9) were applied in all cases and included the following: ≥67% of all QC samples must meet the recovery criteria, and at least 50% of QC replicates at each concentration must meet the recovery criteria.

**Table 3.** Recovery requirements from VICH GL49 (2015)

Analyte concentration	Acceptable range for recovery
<1 µg/kg	50 to 120%
≥1 µg/kg <10 µg/kg	60 to 120%
≥10 µg/kg <100 µg/kg	70 to 110%
≥100 µg/kg	80 to 110%

Table 4 presents the results of the QC samples analyzed for all 10 studies. Unless otherwise noted, QC replicates were tested on one day (within run) for each tissue type. Not all studies examined all tissue types.

**Table 4.** Data summary of QC sample results by tissue

Study No.	Lab No.	Fortification concn, µg/kg	*n*	Recovery or mean recovery, %	Range of recoveries, %	Proportion meeting recovery criterion
Muscle						

1	1	100	6	98.5	94.4–106	6/6
		200	6	89.5	76.5–96.0	5/6
		400	6	81.8	72.5–86.7	4/6
		2000	6	86.5	83.2–88.9	6/6
		4000	6	88.4	86.9–91.9	6/6
		8000	5^a^	88.0	83.5–90.3	5/5
3	3	7.5	1	110	NA	1/1
		15	1	93.5	NA	1/1
		500	1	103	NA	1/1
4	2	4000	3	83.4	77.4–89.6	2/3
9	4	20	6	88.3	87.4–90.5	6/6
		100	6	91.4	89.2–93.5	6/6
		200	6	88.0	85.4–91.4	6/6
		400	6	86.8	85.3–87.9	6/6
10	4	100	13^b^	90.4	84.2–100	13/13
		200	13^b^	89.0	81.7–100	13/13
		400	13^b^	89.6	83.4–100	13/13

Liver						

1	1	100	6	98.5	95.0–101	6/6
		200	6	93.0	91.6–94.2	6/6
		400	6	87.6	86.3–88.7	6/6
		7500	6	95.7	94.7–96.4	6/6
		15* *000	6	97.4	95.5–99.8	6/6
2	2	200	10^c^	95.6	92.7–99.8	10/10
		2000	10^c^	98.4	96.4–102	10/10
		4000	10^c^	99.1	90.1–111	10/10
3	3	25	1	110	NA	1/1
		50	1	99	NA	1/1
		50	1	91.2	NA	1/1
		800	1	109	NA	1/1
4	2	4000	3	78.5	76.2–81.3	1/3
5	2	4000	12^d^	97.1	94.3–101	12/12
6	2	4000	21^e^	98.8	82.6–116	18/21
7	2	4000	2^f^	96.8	94.8–98.7	2/2
8	2	4000	2^f^	96.6	95.7–97.5	2/2
9	4	20	6	104	98.5–108	6/6
		100	6	93.6	92.7–95.5	6/6
		200	6	92.1	90.4–94.1	6/6
		400	6	93.0	92.3–94.2	6/6
10	4	100	10^g^	94.1	88.1–104	10/10
		200	10^g^	90.9	85.9–101	10/10
		400	10^g^	89.5	84.6–100	10/10

Kidney						

1	1	100	6	96.2	93.6–99.5	6/6
		200	6	96.0	95.0–97.6	6/6
		400	6	97.1	96.2–98.8	6/6
		3000	6	97.6	95.9–100	6/6
		6000	6	91.8	69.2–101	6/6
		12* *000	6	97.9	96.4–102	6/6
3	3	7.5	1	87.8	NA	1/1
		15	1	96.3	NA	1/1
		500	1	97.5	NA	1/1
4	2	4000	3	86.6	82.0–89.3	3/3
7	2	4000	1	105	NA	1/1
8	2	4000	2	87.4	86.5–88.3	2/2
9	4	20	6	72.0	69.6–75.5	5/6
		100	6	87.5	84.4–90.9	6/6
		200	6	82.2	80.7–84.0	6/6
		400	6	85.8	84.6–86.9	6/6
10	4	100	12^h^	94.0	83.3–103	12/12
		200	12^h^	92.6	84.1–101	12/12
		400	12^h^	92.5	84.5–101	12/12

Skin with adhering fat						

1	1	100	6	105	104–107	6/6
		200	6	101	98.7–104	6/6
		400	6	95.8	93.6–97.0	6/6
		2000	6	99.7	96.0–102	6/6
		4000	6	102	100.9–104	6/6
		8000	6	97.5	92.3–102	6/6
3	3	25	1	70.9	NA	1/1
		50	1	98.8	NA	1/1
		800	1	99.9	NA	1/1
4	2	4000	3	91.9	91.7–92.3	3/3
9	4	20	6	99.0	95.6–103	6/6
		100	6	94.6	92.6–96.5	6/6
		200	6	91.8	90.6–93.0	6/6
		400	6	91.1	89.2–92.7	6/6
10	4	100	10^g^	99.6	93.7–104	10/10
		200	10^g^	94.7	89.4–102	10/10
		400	10^g^	97.2	91.9–103	10/10

a Outlier removed due to incorrect fortification.

b Represents duplicates tested on each of 2 days followed by triplicates on each of 3 days.

c Represents duplicates tested on each of 5 days.

d Represents triplicates tested on each of 4 days.

e Represents triplicates tested on each of 7 days.

f Represents singlicates tested on each of 2 days.

g Represents duplicates tested on each of 2 days followed by triplicates on each of 2 days.

h Represents duplicates tested on each of 3 days followed by triplicates on each of 2 days.

The grand mean recovery from QC test portion sample analyses across all studies were 91.1% for muscle, 95.8% for skin with adhering fat, 95.1% for liver, and 91.8% for kidney. A few test portion analytical results did not meet the recovery criteria. These were one muscle tissue test portion at 200 µg/kg; two kidney tissue test portions at 400 µg/kg and 6000 µg/kg in Depletion Study 1; two liver tissue test portions at 4000 µg/kg in Depletion Study 4; three liver tissue test portions at 4000 µg/kg in Depletion Study 6; and one kidney tissue test portion at 20 µg/kg in Depletion Study 9. When the additional QC sample criteria from the Bioanalytical Method Validation Guidance for Industry (9) were applied, only one failure was noted, and that was with the 4000 µg/kg liver tissue sample in which two-thirds of the replicates were outside the acceptance range of 80–110% recovery at >100 µg/kg.

The QC data from Table 4 were then analyzed by tissue type for reproducibility among the 10 depletion studies. Since the various studies did not use common fortification concentrations for the QC test portions, the data were analyzed under the following conditions and assumptions: the relative recovery value or mean relative recovery at each concentration was used as the method result; each fortification concentration was treated as one “replicate” (*n *=* *1) in each study; each matrix was analyzed separately; the overall study design was unbalanced since the number of fortification concentrations in each study for each matrix varied from 1 to 6.

Therefore, the data were analyzed in the pC-metamer (pC = −log_10_C, where C is concentration) according to LaBudde (10) using a revised statistical workbook (11).

A summary of the statistical analysis by tissue type is presented in Table 5. Relative standard deviation of repeatability ranged from 5.2 to 8.9%, and relative standard deviation of reproducibility ranged from 6.8 to 8.9%. Thus, both repeatability and reproducibility for all tissue types were well below acceptable precision limits (6). It is interesting to note that for skin with adhering fat the repeatability and reproducibility were equal. Since the reproducibility error comprises error from repeatability and error due to laboratories (in this case studies) with the relationship $s_{r}^{2}$ + $s_{L}^{2}$ = $s_{R}^{2}$, it is meaningful to ascribe the reproducibility error to repeatability error entirely with little or no contribution from laboratory/study error.

**Table 5.** Statistical summary of multi-laboratory data

Tissue	L^a^	$\Sigma n^{b}$	Grand mean recovery^c^, %	RSD(r), % (95% CI)	RSD(R), %	ICC^d^ (95% CI)	Relative recovery^e^, % (95% CI)
Muscle	5	17	91.1	5.4 (3.8, 9.2)	7.9	0.53 (0.00, 1.00)	90.4 (83.8, 97.5)
Liver	10	24	95.1	5.8 (4.1, 9.9)	6.8	0.28 (0.00, 1.00)	94.5 (91.1, 98.0)
Kidney	7	19	91.8	5.2 (3.7, 8.8)	9.0	0.67 (0.26, 1.00)	91.5 (85.3, 98.1)
Skin w/fat	5	17	95.8	8.9 (6.3, 15.1)	8.9	0.00 (0.00, 1.00)	94.5 (89.2, 100.1)

a L = number of laboratory studies. There were a total of four participating laboratories.

b Σn = sum of data points (“replicates”). Each fortification concentration was treated as one replicate in each study.

c Grand mean recovery is the mean of mean recoveries across all concentrations and studies.

d ICC = Intraclass correlation coefficient.

e Relative recovery is the reverse transform of the bias estimate in the pC metamer.

The intraclass correlation coefficient (ICC) is a ratio of the laboratory variance to the reproducibility variance ($s_{L}^{2}$/$s_{R}^{2}$) and can be used to assess laboratory (or study) homogeneity. For example, an ICC value of 0.5 would indicate that the laboratory variance accounts for half of the total reproducibility variance. An ICC value close to 0 indicates a lack of correlation among replicates, meaning that there is no difference among laboratories and replicate results are homogeneous and likely to be normally distributed. While three of the four tissue sample types subjected to chemical analysis yielded point estimates of ICC greater than 0, the confidence intervals are too broad to draw any conclusions. More data sets are needed to reduce the size of the confidence intervals and improve the point estimates of ICC. Since there are currently no requirement or acceptance criteria for ICC, these estimates are provided for informational purposes only. Finally, the relative recovery (calculated as the reverse transform of the bias in the pC-metamer) was 90.4% (95% CI 83.8 to 97.5) for muscle, 94.5% (95% CI 91.1 to 98.0) for liver, 91.5% (95% CI 85.3 to 98.1) for kidney, and 94.5% (95% CI 89.2 to 100.1) for skin/fat, indicating a small bias of results in all matrixes.

### Comments From Participating Laboratories

Comments were solicited from the four laboratories at the conclusion of studies regarding the performance and ease of use of the method. The comments are listed here.

#### Comments/feedback from Laboratory 1

No difficulties were encountered with the technical conduct of the method. Carry-over occurred in a small number of samples. As carry-over is signal dependent, there were some analytical runs where the signal of some reinjected blanks was slightly higher than 20% of the LLOQ. For the following reasons, the carry-over was deemed to have negligible impact on the analyses: it had no effect on calibration curve accuracy, especially for the lower concentrations; it had no effect on QC accuracy; to mitigate the potential impact of carry-over on sample analysis, samples were injected in ascending order of magnitude of expected concentrations based on pre-slaughter withdrawal times. None of the samples with analyte concentration magnitudes approaching the ULOQ were immediately succeeded by samples with concentrations determined at or above the LLOQ, thus indirectly confirming the insignificance of carry-over on the sample analysis; the use of matrix-matched standards and the stable isotope internal standard supported full confidence in the assay results.

The data support consideration of the transition of the method from Official First Action to Official Final Action.

#### Comments/feedback from Laboratory 2

No difficulties were encountered in the technical conduct of the method; the tissue assay results were deemed to reliably reflect the recovery values and calibration plots; the use of matrix-matched standards and the stable isotope internal standard supported full confidence in the assay results.

The data support consideration of the transition of the method from Official First Action to Official Final Action.

#### Comments/feedback from Laboratory 3

No difficulties were encountered with the technical conduct of the method; no carry-over was detected in any of the matrixes; some matrix effect was notable but without significant impact in all four tissue types.

The data support consideration of the transition of the method from Official First Action to Official Final Action.

#### Comments/feedback from Laboratory 4

No difficulties were encountered with the physical performance aspects of the method; the tissue assay results were reliable as reflected in the recovery values and the linearity of the calibration plots; the use of matrix prepared standards and the stable isotope internal standard provide complete confidence in the assay results.

The data support the transition of the method from Official First Action to Official Final Action.

## Conclusions

Nontraditional multi-laboratory data analyzed in the pC-metamer and associated calibration curves are presented to support Final Action status of AOAC Method **2013.07**. The data analysis demonstrated acceptable repeatability and reproducibility with a very small bias observed in the accuracy of the method.
